# A method to measure the partitioning coefficient of volatile organic compounds in nanoparticles

**DOI:** 10.1016/j.mex.2020.101041

**Published:** 2020-08-22

**Authors:** Guiying Rao, Jeonghyeon Ahn, Abigail Evans, Michelle Casey, Eric Vejerano

**Affiliations:** Center for Environmental Nanoscience and Risk, Department of Environmental Health Sciences, University of South Carolina, 921 Assembly St., PHRC 501D, Columbia 29208, United States

**Keywords:** Gas/particle partitioning, VOCs, Nanoparticles, Aerosols, Methods

## Abstract

The partitioning behavior of volatile organic compounds (VOCs) into nanoparticles is less studied compared to those of semivolatile organic compounds (SVOCs) because of the lower concentration of the VOCs that is expected to partition into particles. One challenge in measuring the accurate partition coefficient of VOCs is quantifying their low mass fraction that sorbed on nanoparticles and differentiating them from the high VOC concentrations present in the gas-phase. Systematically characterizing the partitioning coefficient at a specific environmental condition is also difficult when sampling in the field. During field sampling, thermal and non-thermal issues such as sampling artifacts and non-equilibrium conditions because of a dynamic environment often result in considerable variability in the measured partition coefficients. In this study, we developed a bench-scale system that can achieve precise control of the experimental condition (*e.g.,* relative humidity, temperature, and particle composition) and allow us to measure the low concentration of 1,2-dichlorobenzene in the particles. A similar set up can be used to study the low mass fraction of other VOCs partitioning in nanoparticles. The detailed but uncomplicated system setup may assist other researchers that investigate the global fate and transport and health effects of VOCs.

• A bench-scale system was built in the laboratory to study the gas-to-particle partitioning
• Experimental conditions can be controlled and easily varied
• The system enables the systematic study of a single environmental factor on the partitioning process

## Graphical abstract

**Figure:**
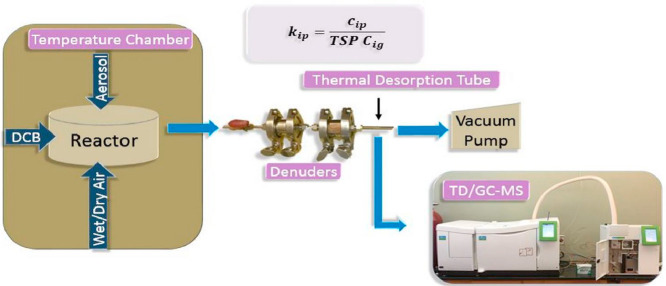

Specifications table

**Table:** 

Subject area:	*Select one of the following subject areas:* • *Environmental Science*
More specific subject area:	Atmospheric pollution by VOCs and aerosol nanoparticles
*Name and reference of original method:*	Direct submission of a new method
Resource availability:	Software: http://www.ni.com/en-us/shop/labview/download.html Hardware: http://www.perkinelmer.com/product/turbomatrix-100-td-m0413651 http://www.perkinelmer.com/category/gas-chromatography-mass-spectrometry-gc-ms Reagent: https://www.sigmaaldrich.com/united-states.html https://www.fishersci.com/us/en/brands/I8T3NQD9/fisher-chemical.html

## Method details

 

## Materials

• Anhydrous 1,2-dichlorobenzene (1,2-DCB) (99%) from the Sigma-Aldrich
• Ammonium sulfate ((NH_4_)_2_SO_4_) (≥99%) from the Sigma-Aldrich
• 1,2-Dichlorobenzene-d4 (1,2-DCB-d4, 0.2 mg/mL in methanol, >99.99%) from the AccuStandard
• Methanol (>99.99%) from the Fisher Chemical

## Equation for calculating the gas/particle partitioning coefficient (*K_ip_*) [1]

(1) $$K_{ip}=\frac{C_{ip}}{C_{ig}TSP},m^{3}/\mu g$$

where *C_ip_* and *C_ig_* are the concentrations of the VOC, *i,* in the aerosol and gas phase, respectively (µg/m^3^ or ppb, expressed in the same units for *C_ip_* and *C_ig_*), and TSP is the mass concentration of the dry aerosols (µg/m^3^). At 25 °C, the concentration of 1,2-DCB (ppb)=0.166 · *C_ip_* or *C_ig_* in µg/m^3^.

## Step 1: Setting up the experimental system

**Fig. 1:**
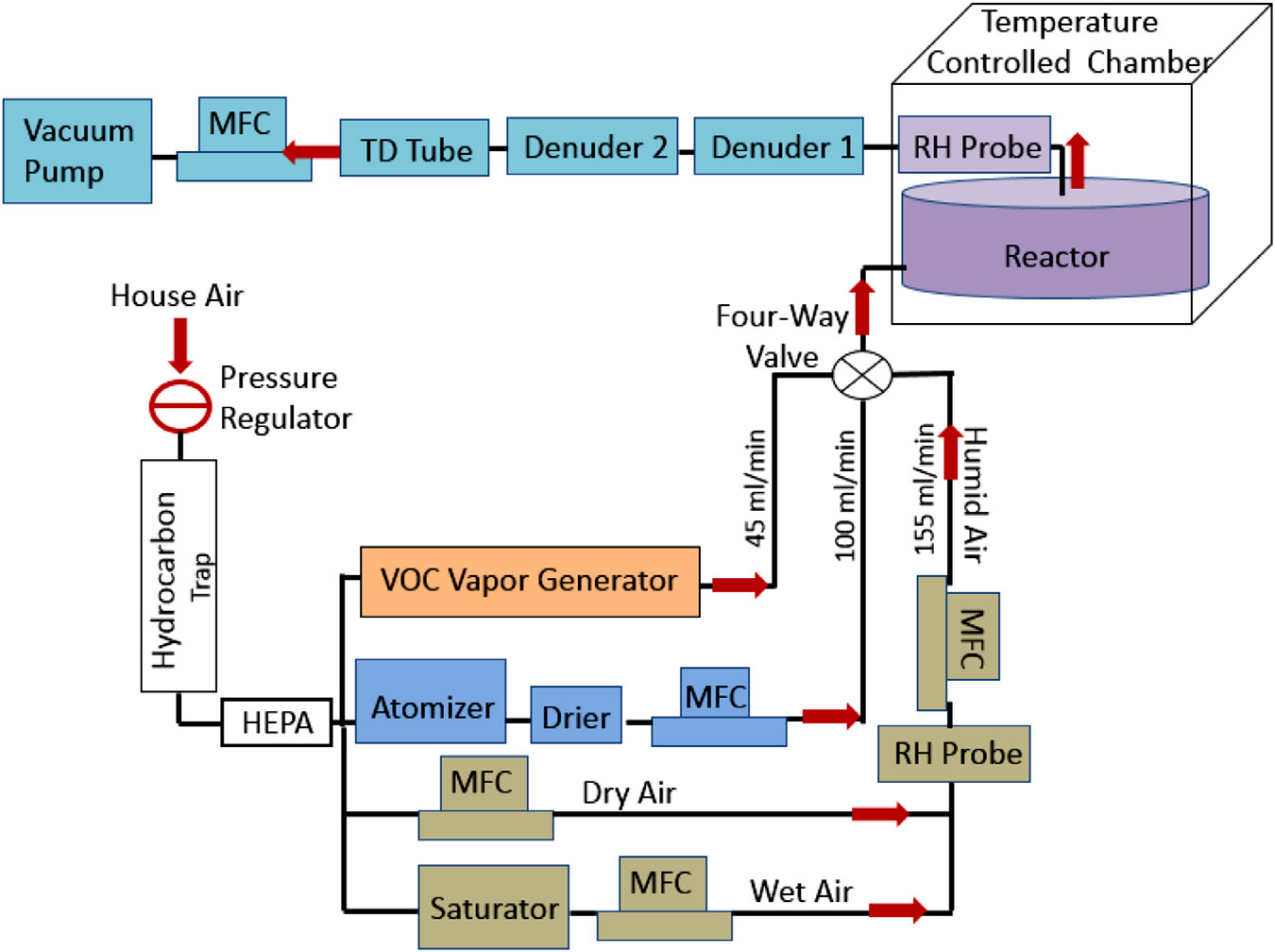
Experimental setup diagram used to examine the gas-to-particle partitioning of VOCs under various environmental conditions.

**Fig. 2:**
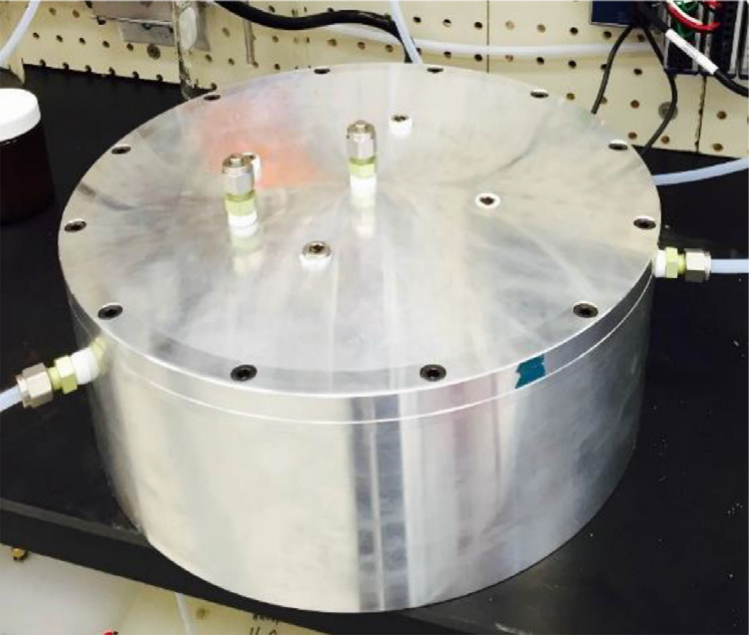
Aluminum environmental chamber used for experimentation.

Traditional methods to sample for atmospheric nanoparticles that contain VOCs are performed in the field [2,3]. During sampling, precisely controlling the environmental factor is important to measure the accurate partition coefficient of VOCs. Here, a laboratory system was built to measure the accurate partition coefficient of a surrogate VOC, 1,2-dichlorobenzene (Fig. 1). Three flow streams, namely, the (1) diluted 1,2-DCB, (2) aerosol, and (3) the humid air flow streams, were connected to the inlet of a chamber that were enclosed in a temperature-controlled cabinet (CEO932, Lunaire Environmental, New Columbia, PA) to maintain a constant temperature. An aluminum environmental chamber was fabricated (Fig. 2), measuring 0.3 m in diameter and 0.1 m in height. We used aluminum to minimize the adsorption of VOCs and nanoparticles to the chamber's surface. The environmental chamber had a removable lid that attached to the base using 12 Allen screws. An *o*-ring rested in the groove on the top rim of the chamber base to ensure a tight seal between the lid and base. We supplied compressed house air at a pressure of 240 kPa as our air source, which we passed through a trap to remove hydrocarbons (BHT-4, Agilent) and through a high-efficiency particulate air (HEPA) filter (83690H, Pall Life Sciences) to remove particles. Hereafter, house air refers to clean and particle-free air.

The dilute 1,2-DCB flow stream was generated by placing a 2-mL vial containing 1 mL of pure 1,2-DCB liquid in the chamber of the Precision Standard Gas Generator (491M-B, KIN-TEC Laboratories Inc, La Marque, TX). Pure DCB vapor effused through a 400-µm deactivated glass capillary, which we diluted with the purified house air. By adjusting the flow rate of the air stream passing through the chamber of the gas generator, we can set the concentration of the diluted 1,2-DCB. Here, 45 mL/min of particle-free air diluted 1,2-DCB resulting in a dilute 1,2-DCB concentration of 17±1 ppbv.

The aerosol flow stream of nanoparticles was generated by atomizing a 250 ppm (NH_4_)_2_SO_4_ solution as the particle precursor. We operated the atomizer (TSI3076, TSI Incorporated, Shoreview, MN) at 2.5 L/min of house air and 207 kPa. A mass flow controller (MFC) (FC-280, Tylan) interfaced with a LabVIEW program that controlled the flow rate of the house air. By varying the concentrations of the precursor, we can generate aerosols with different particle number concentrations and size distributions. The aerosol stream was passed through a diffusion dryer (Model 3062, TSI Incorporated, Shoreview, MN) containing silica as the desiccant to remove water in the aerosol stream and those bound on the particles. A needle valve connected to a tee (with one end open to the atmosphere) was installed on the aerosol stream to allow 100 mL/min of aerosols to flow into the chamber. A needle valve instead of a mass flow controller (MFC) was used to minimize fluctuations in the concentration and particle size of the aerosols.

The humid air stream was generated by mixing the dry house air (relative humidity (RH) ~4%) with wet air. We prepared the wet air using two 20-gallon saturators filled with water that were connected in series. To prevent water from condensing in the MFC and damaging it, before mixing of the wet air and the dry air, an empty 1-L glass container was placed between the saturator and the MFC. The desired RH was generated by adjusting the flow rates of the dry air and the wet air. We measured the RH using a temperature and humidity probe (USBQTENKI-T-RH-CC2, Dracal Technologies, Inc.) that was coupled with the LabVIEW program for control of RH and for monitoring the chamber temperature. Here, we set the wet air flow rate to 155 mL/min, and the total flow rates for all three flows to 300 mL/min (aerosol flow: 100 mL/min; DCB flow: 45 mL/min) entering into the chamber resulting in 20% RH. The flow rates of the dry air, wet air, and the air flowing into the chambers were all controlled by the LabVIEW program coupled with the MFCs. If other flow rates are preferred, one can simply adjust them in LabVIEW.

For sampling the aerosols that contained 1,2-DCB, we pulled the air stream in the chamber using a vacuum pump (Model 10473, Environmental Monitoring System) coupled to an MFC at a flow rate of 300 mL/min. We used a Tenax packed-thermal desorption (TD) tube (Markes International, Inc, Gold River, CA) to trap the aerosols. The sampled gas stream first passed through two multi-channel carbon denuders (ADI-DEN2, Aerosol Dynamics Inc., Berkeley, CA) connected in series to remove the gas-phase 1,2-DCB. The carbon denuder is 20 cm long, which is suitable for removing VOCs at flow rates up to 5 L/min for PM_2.5_ sampling. Each adsorption monolith is composed of pure carbon without binders containing approximately 500 channels and a specific surface area of ~700 m^2^/g. The monolith is permanently sealed inside a 302 stainless steel housing. Before each sampling, we conditioned both denuders at 135 °C for 10 h using high-purity nitrogen gas (purity > 99.999%) at a flow rate of 150 mL/min in each. Preliminary results demonstrated that the denuders were highly efficient for adsorbing 1,2-DCB vapor in the air; of the ~1000 µg of 1,2-DCB vapor that entered the denuders only ~50 pg were detected in the outlet. Also, at 20% RH, ~90% of the aerosols passed through the denuder.

## Step 2: LabVIEW/MFC setup

**Fig. 3:**
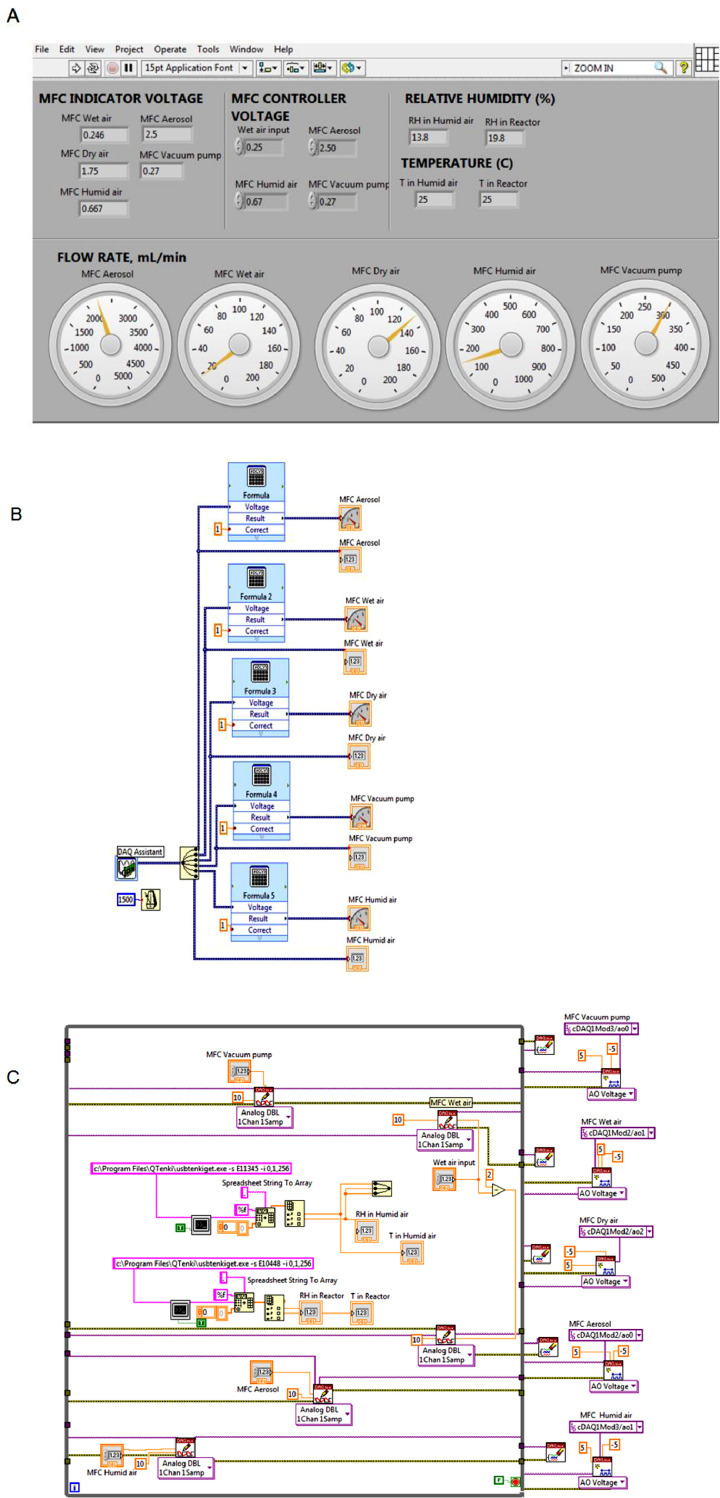
The front panel (A) and block diagram (B & C) of the LabVIEW program.

A custom-built LabVIEW Virtual Instrument (VI) control interface was built and verified for operational use. Inputs into the LabVIEW VI allowed for management of MFCs and the RH probe. Communication of the LabVIEW VI with the MFCs and RH probe controlled the flow rates of humid air stream of specific RH value and the sampling flow rate. RH and flow rates can be controlled and monitored in the LabVIEW VI program. Fig. 3 depicts the LabVIEW profiles of the front panel and the block diagram. We have also included the complete NI LabVIEW files for the program. The Supplementary Material depicts the detailed block diagram, which can be viewed by downloading the NI LabView software available from National Instruments (https://www.ni.com/en-us.html). If necessary, the RH probe provides feedback for controlling the MFC using a virtual PID controller in LabVIEW.

## Step 3: TD-GC/MS setup for VOC quantification

For quantifying the amount of VOCs in the TD tubes, we used a thermal desorber (TurboMatrix 100, PerkinElmer, Waltham, MA) coupled to a gas chromatograph (Clarus 680, PerkinElmer, Waltham, MA) and mass spectrometry (Clarus SQ8T, PerkinElmer, Waltham, MA) (TD-GC/MS). The capillary GC column (Agilent DB-5ms) has a length of 30 m, an inner diameter of 0.25 mm, and a film thickness of 0.25 µm coated with 5% (phenyl)-methylpolysiloxane. We first thermally desorbed the TD tubes containing 1,2-DCB, and then identified the compound based on its retention time (4.63 min) and abundant ions in the mass spectrum. Spectra were acquired using selected ion monitoring (SIM) mode to increase the sensitivity for detecting and quantifying trace levels of 1,2-DCB using the *m/z* ratios of 111 and 146. We used 1-µL of deuterated 1,2-DCB (1,2-DCB-d4) as the internal standard (IS) at a concentration of 500 ppb. IS was injected to the sorbent bed on the TD tube for quality control in the TD-GC/MS analysis. We quantified only the abundant ions that are unique to the IS (*m/z* ratios of 78, 115, and 152) to discriminate it from 1,2-DCB. The parameters used in the TD-GC/MS system are summarized in Table 1.

**Table 1:** Parameters used for TD-GC/MS analysis of 1,2-DCB and 1,2-DCB-d4.

Parameter	Value
**Thermal desorber**	
*Primary desorption (Tenax TD tube)*	
Valve temperature	225°C
Tube temperature	225°C
Transfer line temperature	250°C
Trap temperature	-30 to 300°C, heating rate of 40°C/s
Purge time	1 min
Desorption time	5 min
Trap hold time	7 min
Desorption gas	Helium (99.999%)
Desorption flow	50 mL/min
Inlet split flow	20 mL/min
Outlet split flow	20 mL/min
Column pressure	12 psi
Trap packing material	Tenax TA
**GC/MS**	
*GC Oven*	
Temperature	50°C for 0.5 min, 25°C/min to 100°C,
	180°C, hold for 1 min
GC column flow	1 mL/min
Carrier gas and flowrate	Helium (99.999%), 1mL/min
*MS (detector)*	
Electron Energy	70
Trap Emission	100
Repeller	1.0
Lens 1, 2	5, 55
Filament current	0.07
Source temp	200°C
LM Res	8.0
HM Res	12.5
Ion Energy	2.5 (ramp of 1.5)
Multiplier	1650 V
*SIM (detection method)*	
m/z of 1,2-DCB	111, 146
m/z of 1,2-DCB-d4	78, 115, 152
Dwell time	0.05 seconds
Ionization mode	EI+
Inter-channel delay	0.05
Retention window	4.3 to 5 min
Solvent delay	4 min

**Fig. 4:**
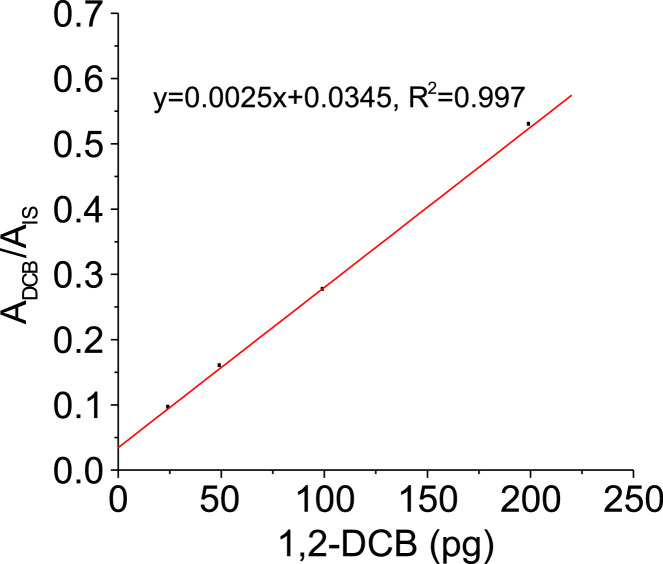
The calibration curve for 1,2-DCB quantification by TD-GC/MS.

1,2-DCB was quantified in each test using a calibration curve. To generate the calibration curve, we prepared 0, 25, 50, 100, and 200 ppb of 1,2-DCB solution in a 2-mL amber GC vials using methanol as the solvent. IS (500 ppb) was added on each vial. Next, we injected 1 µL of each solution into a clean TD tube and analyzed it using TD-GC/MS. To generate the calibration curve, we plotted the ratio of the peak areas of 1,2-DCB (A_DCB_) to that of 1,2-DCB-d4 (A_IS_) with the mass of the compound as shown in Fig. 4. We estimated the limit of detection to be 13 pg, which we calculated as the ratio of 3 × the standard deviation (0.0108) of the low concentration sample to the slope of the calibration curve (0.0025). The calibration curve represents the lower part of the dynamic range for the range of concentration of 1,2-DCB expected to partition into nanoparticles. For VOC partitioning, working in the lower region of the dynamic range is desired. Increasing the sampling time will increase the breakthrough of 1,2-DCB from the substrate, even more so for highly volatile compounds, leading to a higher error in the measured partition coefficient.

## Step 4: Measuring *K_ip_*

To obtain *K_ip_*, according to Eq. (1), we characterize the 1,2-DCB concentrations in the gas phase (*C_ig_*) and those in the aerosols (*C_ip_*) as well as the mass concentration of the aerosols (TSP) We calculate *C_ig_* as:

(2) $$C_{ig}=\frac{m_{ig}}{V},\mu g/m^{3},\text{ppb}$$

where *m_ig_* is the total mass of 1,2-DCB inside the chamber, and *V* is the volume of the air that we sampled. To obtain *C_ig_*, a Tenax-packed TD tube was used to collect the air flowing out of the chamber before it entered the denuders (Fig. 1). The sampling flow rate was kept at 25 mL/min using a vacuum pump with a total sampling time of 0.5 min, corresponding to a *V* of 12.5 mL. Preliminary test suggests that one TD tube was sufficient to capture 1,2-DCB. We quantified 1,2-DCB as describer earlier. Because the concentration of 1,2-DCB in the gas phase was several orders of magnitude greater than those in aerosols (consistent with what observed in the field [4,5]), the quantified 1,2-DCB mass was used as *m_ig_* without deducting the mass of 1,2-DCB in the aerosols. A *C_ig_* of 2.57±0.12 ppb was characterized in the chamber, which was consistent with the 17 ppb concentration in the diluted 1,2-DCB flow stream before entering the chamber.

**Fig. 5:**
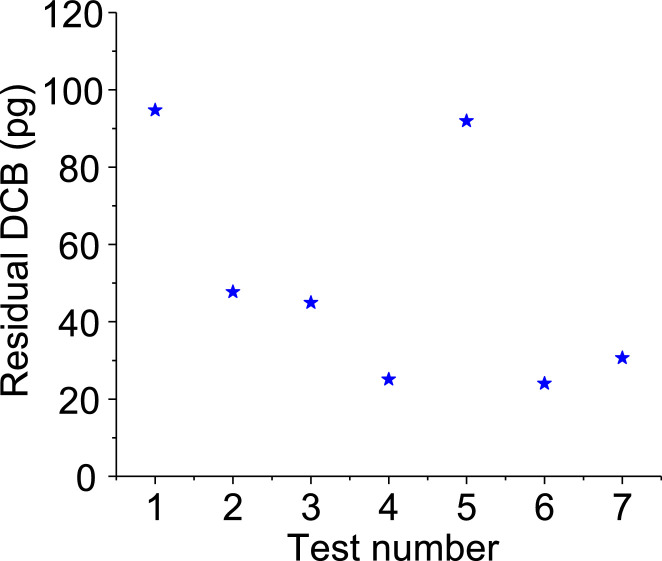
Control test results for measuring the residual 1,2-DCB mass that exited from the two denuders.

Similar to *C_ig_*, the *C_ip_* was calculated as:

(3) $$C_{ip}=\frac{m_{ip}}{V},\mu g/m^{3},\text{ppb}$$

where *m_ip_* is the total mass of 1,2-DCB that partitioned into the aerosols in the sampled gas volume *V*. The total sampling time was set for 24 h, corresponding to a sampling volume of 0.432 m^3^ in each test. After sampling, the 1,2-DCB in the TD tube was desorbed in the TD-GC/MS system for quantification. Because the denuders may not completely adsorb the 1,2-DCB in the gas stream, residual 1,2-DCB coming out the denuders will be adsorbed by the succeeding TD tube, leading to a higher quantified *m_ip_*. Thus, we performed control tests without aerosols in the system as described in Fig. 1 to measure the mass of the residual 1,2-DCB that was not adsorbed by the denuders. In these tests, the 100 mL/min of aerosol flow was replaced by 100 mL/min of clean house air, and we adjusted the RH by varying the wet to dry air ratio. The results are given in Fig. 5. We measured a residual 1,2-DCB mass of 51±32 pg. This value was found to be insensitive to the RH or temperature in the chamber. We deducted 51 pg from the 1,2-DCB mass analyzed by the TD-GC/MS to obtain *m_ip_*. Although 51 pg is minimal compared to the total amount of 1,2-DCB entering into the denuders (2.57 ppb × 0.432 m^3^ = 1110 µg), the picogram level of residual 1,2-DCB cannot be neglected in calculating the *K_ip_* because the measured *m_ip_* for the 24-h sampling test is in similar order.

**Fig. 6:**
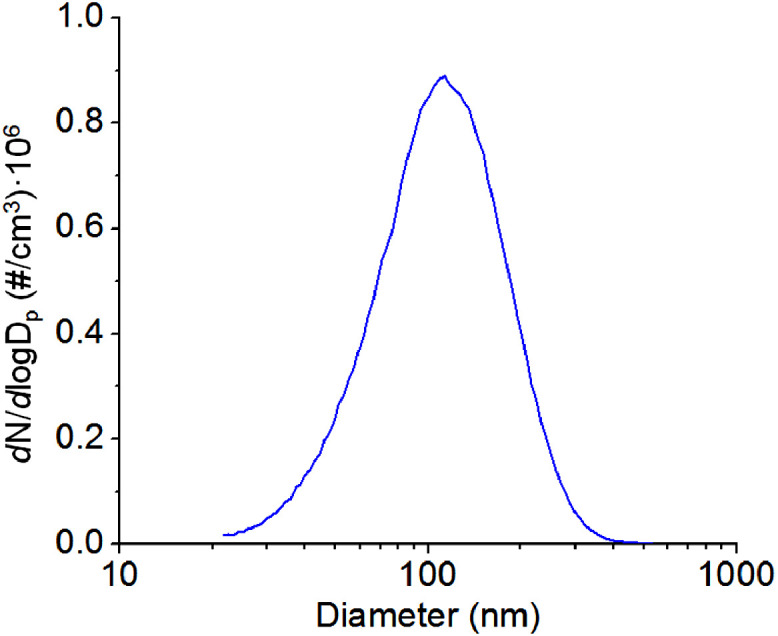
Particle size distribution of dry aerosols after exiting the chamber.

The TSP of the sampling gas was characterized by an Electrostatic Classifier (Model 3082, TSI Incorporated, Shoreview, MN) and Condensation Particle Counter (Model 3775, TSI Incorporated, Shoreview, MN). The 1,2-DCB flow stream in Fig. 1 was replaced by house air at the same flow rate to remove the effect of the adsorbed 1,2-DCB on the TSP of the pristine aerosols (absence of 1,2-DCB). (NH_4_)_2_SO_4_ nanoparticles are highly hygroscopic [6]. To measure the TSP of the “dry” aerosols and reduce the effect of absorbed water on TSP, the RH in the chamber was controlled to the minimum achievable RH in the lab, which is an RH of ~4%. The outlet of the chamber was connected to the TSI instrument and measured at a flow rate of 300 mL/min. A typical result in one measurement is shown in Fig. 6. At least five measurements were performed, leading to an average TSP of 1186±92 µg/m^3^, a particle median diameter of 107 ± 1 nm, and a particle number concentration of (4.66±0.34) × 10^5^/cm^3^.

Aerosols flowing out of the chamber may deposit in the denuders or may not be retained completely by the TD tubes, leading to a loss of TSP during sampling. Preliminary test showed a higher deposition and retention of aerosols by the denuders and TD tube at a higher RH in the chamber. Therefore, aerosols at the outlet of the chamber, denuders, and TD tube (Fig. 1) were each characterized to identify the deposition efficiency in the denuders and TD tube at a specific RH. A 20% RH in the chamber was studied here. Results are given in Table 2. As can be seen, the TSP of 1287±64.4 µg/m^3^ at RH of 20% is greater than the TSP of the dry aerosols (1186.5±92.5 µg/m^3^), suggesting the adsorption of water molecular by the aerosol nanoparticles. Only ~9% of aerosols were deposited in the denuders while the TD tube trapped 86% of the following aerosol nanoparticles; thus the current system effectively retained 78% of the aerosols coming out of the chamber, corresponding to a dry aerosol TSP of 930 µg/m^3^.

**Table 2:** Aerosol characteristics at each sampling point and the deposition efficiencies in the denuders and TD tube at RH of 20%.

Sampling position	Particle median diameter (nm)	TSP (µg/m^3^)	Particle number concentration (× 10^5^, #/cm^3^)	Deposition efficiency^a^ (%)
Outlet of chamber	112±4	1287±64	4.55±0.30	n/a.
Outlet of denuders	114±2	1172±28	4.00±0.10	9% in denuders
Outlet of TD tube	146±11	166.7±58	0.34±0.10	86% in TD tube

a calculated based on the TSP data. n/a: not applicable).

## Method validation

**Fig. 7:**
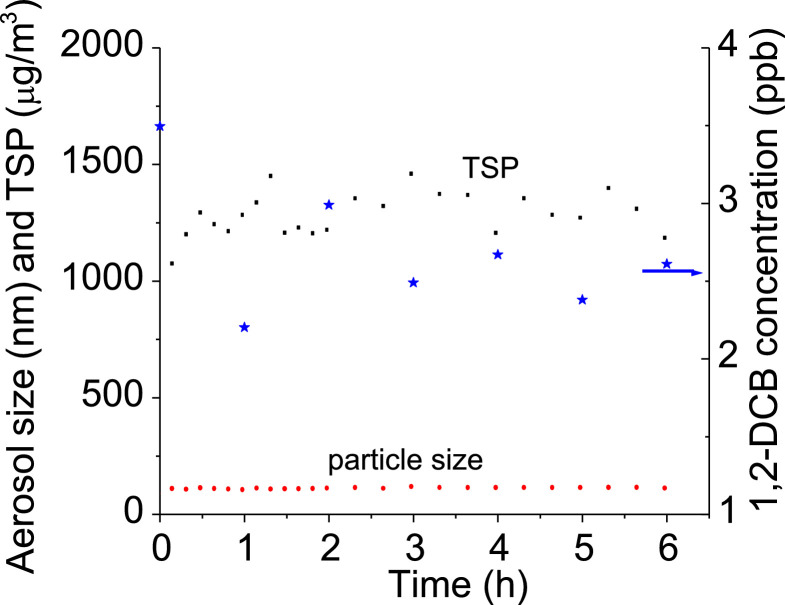
Evolution of aerosol particle size (red marker), TSP (black marker), and 1,2-DCB concentration (blue marker) with time at 20% RH and 25°C.

The experiments were performed at 20% RH and temperatures of 15 and 35 °C, respectively. Before sampling, the 1,2-DCB, aerosols, and humid air all continuously flowed into and out of the chamber for at least 2 h at constant flow rates (45, 100, and 155 mL/min, respectively) to reach a stable experimental condition and the adsorption-desorption equilibrium. The aerosol properties (particle size and TSP) and 1,2-DCB concentration coming out of the chamber were monitored over time to identify the length of time it takes for the system to stabilize. Fig. 7 suggests that 2 h is sufficient for the system to stabilize.

**Fig. 8:**
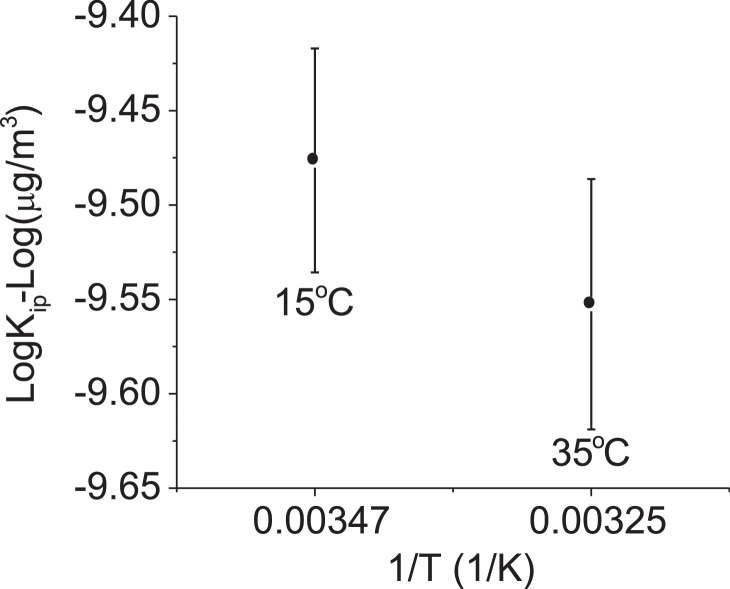
Correlating results of 1/T to log*K_ip_* for tests at 20% RH and temperatures of 15 and 35 °C.

The TD tube was pre-conditioned at 330 °C for 20 min using ultrapure nitrogen gas at a flow rate of 50 mL/min. After that, it was tested in the TD-GC/MS system after conditioning, and again before sampling to reconfirm the absence of 1,2-DCB contaminants. The *K_ip_* results are shown in Table 3 and Fig. 8. The *K_ip_* values ranged from 2.80 × 10^−10^ to 3.34 × 10^−10^ m^3^/µg and showed a negative correlation with temperature, which is consistent with the classical van't Hoff equation [7,8]. For 1 µg of aerosol particles, 0.72 to 0.85 pg of 1,2-DCB partitioned into them. This mass fraction is in similar order (0.1 pg) to those reported in the literature [4], which used urban aerosols, in which the atmosphere contained much lower concentrations of 1,2-DCB and aerosols.

**Table 3:** Calculated 1,2-DCB-to-aerosol partitioning coefficient (*K_ip_*) and the amount of 1,2-DCB partitioned in 1 µg of aerosols at an RH of 20%.

T (°C)	*K_ip_* × 10^−10^ (m^3^/µg)	pg_DCB_/µg_aerosol_
15	3.34±0.585	0.85±0.14
35	2.80 ±0.409	0.72±0.10

## Declaration of Competing Interest

We declare no competing interest.

## Appendix Supplementary materials

Image, application 1
